# Correction to: Administration of gamma‐hydroxybutyrate instead of beta‐hydroxybutyrate to a liver transplant recipient suffering from propionic acidaemia and cardiomyopathy: a case report on a medication prescribing error.

**DOI:** 10.1002/jmd2.12136

**Published:** 2020-09-01

In this paper,^1^ one of the authors, Joel Schlatter was listed incorrectly as Joel Schlattler.

Therefore, the correct author list is:

Caroline Tuchmann‐Durand, Eloise Thevenet, Florence Moulin, Fabrice Lesage, Juliette Bouchereau, Mehdi Oualha, Diala Khraiche, Anaïs Brassier, Camille Wicker, Stéphanie Gobin‐Limballe, Jean‐Baptiste Arnoux, Florence Lacaille, Clotilde Wicart, Bruno Coat, Joel Schlatter, Salvatore Cisternino, Sylvain Renolleau, Philippe‐Henri Secretan, Pascale De Lonlay.
